# Topically Applied Magnetized Saline Water Activates Autophagy in the Scalp and Increases Hair Count and Hair Mass Index in Men With Mild-to-Moderate Androgenetic Alopecia

**DOI:** 10.7759/cureus.49565

**Published:** 2023-11-28

**Authors:** Ángel García Martín, Andrés Santiago Sáez, Manuel Gómez Serrano, Miryam Liaño Riera, Piercarlo Minoretti

**Affiliations:** 1 Legal Medicine, Psychiatry and Pathology, Complutense University of Madrid, Madrid, ESP; 2 Legal Medicine, Psychiatry and Pathology, Hospital Clinico San Carlos, Madrid, ESP; 3 General Direction, Studio Minoretti, Oggiono, ITA

**Keywords:** autophagy, hair mass index, hair count, men, androgenetic alopecia, magnetized saline water

## Abstract

Introduction

Water is essential for life and is vital for almost all functions of the human body. Recent studies have shown that treating water with magnets can alter its physicochemical properties, including intracluster bonds and water-ion interactions. Magnetized water also undergoes modifications in its physicochemical characteristics, such as pH, salinity, and dissolved oxygen. While there is a significant amount of literature on the use of magnetized water in agricultural settings, research on its potential biomedical applications is still limited. Based on previous findings indicating a potential relationship between autophagy activation and hair loss reversal, a pilot study was designed to explore the effects of topically applied magnetized saline water in patients with androgenetic alopecia. The hypothesis was that the process of water magnetization, which promotes the creation of hydroxyl ions, could potentially induce hair growth through the induction of alkali-induced autophagy in the scalp.

Methods

We recruited 20 Caucasian men with mild-to-moderate androgenetic alopecia (Norwood-Hamilton stages II−III). Initially, we conducted a 12-week open-label study to evaluate the potential of a topical lotion containing 95% magnetized saline water (2 mL applied once daily) to increase hair count and hair mass index (HMI). Subsequently, we investigated the effect of the lotion on two autophagy markers (Beclin-1 and LC3B) in scalp biopsies from a subgroup of 10 men.

Results

Hair count significantly increased after 12 weeks of topical treatment with magnetized saline water (from 20.6 ± 9.8 at baseline to 32.5 ± 12.4 at 12 weeks, P < 0.001). Similarly, the mean HMI increased from 37.8 ± 11.4 at baseline to 45.1 ± 13.6 at 12 weeks (P < 0.01). At the molecular level, the topical lotion effectively increased Beclin-1 levels in scalp biopsies by 44% at 12 weeks as compared to the baseline. Similarly, LC3B levels increased by 36% from baseline to 12 weeks, indicating that the lotion effectively activated autophagy in the scalp.

Conclusions

After 12 weeks of topical treatment, a lotion containing magnetized saline water activated scalp autophagy and significantly increased hair count and HMI in men with mild-to-moderate androgenetic alopecia.

## Introduction

Androgenetic alopecia is a common cause of hair loss, affecting approximately half of men and women over the age of 40 in the Caucasian population [1,2]. Traditionally, the main factors contributing to androgenetic alopecia are the increased activity of 5α-reductase in hair follicles and genetic predisposition [3]. Despite extensive research, the only FDA-approved treatments for androgenetic alopecia are topical minoxidil, oral finasteride, and low-level light therapy [4]. Therefore, additional products are commonly used in combination with these approaches to enhance their efficacy and improve response rates [5].

Autophagy is a bulk degradation pathway that helps maintain cellular homeostasis through the removal of obsolete proteins and damaged organelles [6]. It not only helps maintain intracellular balance but also plays a crucial role in various physiological and pathological processes, including hair growth [7]. Small molecules that activate autophagy, such as α-ketoglutarate, α-ketobutyrate, rapamycin, and metformin, have been found to stimulate hair follicles to initiate anagen [8]. This stimulation is blocked by specific autophagy inhibitors, indicating a mechanistic link between autophagy and hair regeneration [8]. In addition, inhibition of autophagy has been shown to lead to premature hair follicle regression and slower hair growth [9]. Interestingly, hydroxytyrosol, a primary phenolic antioxidant found in olive oil, has been demonstrated to increase the secretion of hair growth factors in rat dermal papilla cells by inducing autophagy [10]. These findings suggest that autophagy plays a crucial role in hair growth and regeneration, and its activation can be a potential target for the treatment of hair loss.

When water is exposed to a magnetic field, it undergoes several notable changes in its properties [11]. These modifications include an increase in electrical conductivity, lowered density, reduced surface tension, and a rise in alkalinity [12]. Although research on its medical applications is still limited, a significant body of evidence has been published on the effects of magnetized saline water, which is typically alkaline [11,12], on both plants [13,14] and animals [11]. For instance, studies have shown that when aquatic animals are exposed to saline alkaline water, there is a dose-dependent increase in the expression of autophagy-related genes [15]. In addition, animal studies suggest that magnetized water may have promising potential in a range of medical applications [16-18].

Here, we hypothesized that the topical application of magnetized saline water in the form of a lotion could trigger autophagy, potentially promoting hair growth in men affected by androgenetic alopecia. To validate our hypothesis, we initiated two distinct substudies. The first involved a 12-week, uncontrolled, open-label investigation designed to evaluate the efficiency of magnetized saline water in enhancing hair density and hair mass index (HMI) in male androgenetic alopecia [19]. The second substudy delved into the molecular effects of magnetized saline water on autophagy. To this aim, post-treatment scalp biopsies were probed for the expression levels of two key indicators of autophagy, Beclin-1 and LC3B [20].

## Materials and methods

Design and participants

Twenty Caucasian men aged between 21 and 63 years, all of whom had mild-to-moderate androgenetic alopecia (Norwood-Hamilton stages II−III) [21], were enrolled in a 12-week open-label pilot study. All procedures were performed in outpatient facilities belonging to Studio Minoretti Srl (Oggiono, Italy). Individuals under 18 years of age, having undergone low-level light therapy, follicular transplantation, or surgical interventions for androgenetic alopecia, stem cell therapy, suffering from significant physical illnesses, and having nonpatterned or cicatricial alopecia were excluded, as were those having used finasteride, topical minoxidil, topical prostaglandins, or prostamide in the past 12 months. Throughout the 12-week treatment period, participants were not allowed to use any other topical or systemic active agent for androgenetic alopecia, including food supplements. The study received approval from the local ethics committee (Studio Minoretti reference: SM/AQL/23), and all participants provided written informed consent.

Research product

The research product was a lotion comprising 95% magnetized saline water derived through an exclusive patented method (Aquavis Srl, Brescia, Italy), serving as the only active ingredient. The lotion was sourced directly from the manufacturer. Participants were instructed to apply about 2 mL of the product directly to the vertex area of the scalp once a day in the evening for 12 weeks. To ensure optimal absorption, the solution was to be applied to clean, dry hair, and if necessary, participants were encouraged to part their hair to expose the scalp. They were also advised to gently massage the solution throughout the area of hair loss for comprehensive coverage. 

Clinical study endpoints

The clinical study aimed to assess changes in hair count and HMI over a 12-week period, serving as the primary endpoints. Secondary objectives included evaluating tolerance and overall satisfaction with the topical lotion after 12 weeks. Hair count was measured before and after treatment using the TrichoScan method (Tricholog GmbH, Freiburg, Germany), following the manufacturer's instructions [22]. The recorded photographs were analyzed using TrichoScan software (Version 3.0), and the results were expressed as the number of detected hairs. The HairCheck® System (Divi International Co., Miami, FL, USA) was used to evaluate the HMI, a metric that takes into account both hair density and diameter [19]. To assess tolerance, participants were closely monitored for any local discomfort as well as any systemic adverse reactions. Participants also provided feedback on their overall satisfaction with the lotion, rating it as excellent, good, average, or poor.

Molecular substudy endpoints

In a molecular substudy, a subset of ten patients volunteered to undergo paired 4-mm^2^ punch biopsies from their temporal scalp before and after the 12-week treatment period. Our primary objective was to determine whether the application of the lotion could effectively induce autophagy in the scalp. The collected tissue was thoroughly homogenized, and we used two commercial ELISA kits (MyBioSource Inc., San Diego, CA, USA) to analyze the concentrations of Beclin-1 and LC3B in scalp homogenates. To determine the concentrations of each biomarker, we created standard curves and converted the mean fluorescence intensity from each well into a concentration using the linear part of the standard curve. The results were expressed in arbitrary units (a.u.), with 100 as the baseline value before treatment. We performed each measurement twice and averaged the results. The intra-assay and inter-assay coefficients of variation were below 8% and 10%, respectively.

Data analysis

This is an exploratory study, and no formal power calculation was originally performed. Descriptive statistics were used to summarize the variables of interest. To compare pre- and post-treatment data, paired Student’s t-tests were employed. The Spearman’s correlation coefficient (rho) was applied to test the correlation between the study variables. Analyses were performed using IBM SPSS Statistics for Windows, Version 20 (released 2011; IBM Corp., Armonk, New York, United States) and GraphPad Prism, Version 7.0 (GraphPad Inc., San Diego, CA, USA). A two-tailed P value of <0.05 was considered statistically significant.

## Results

Clinical study

**Figure 1:**
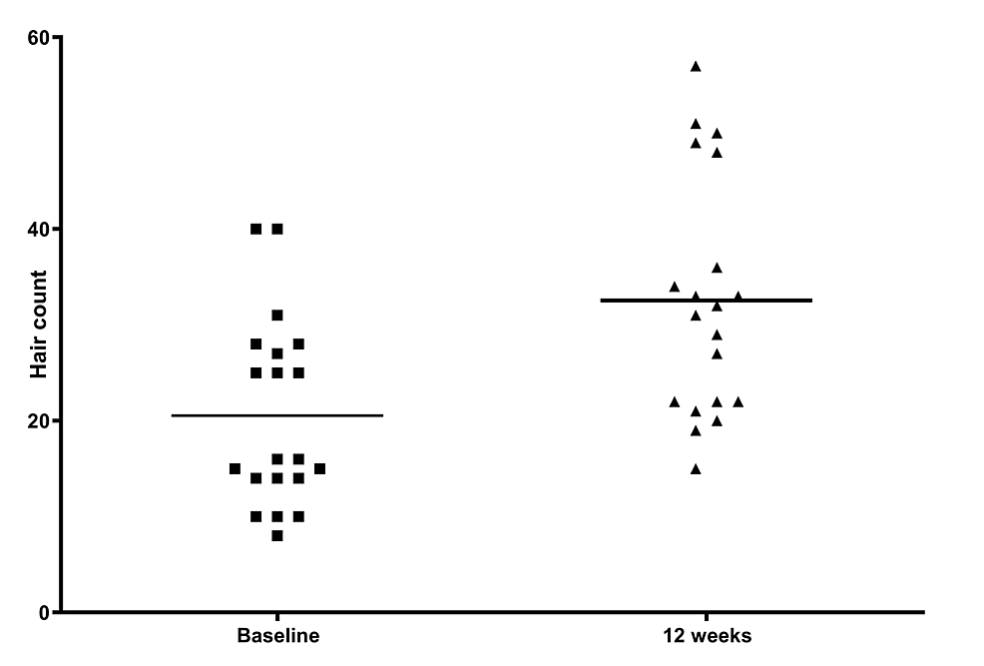
Scatter plots depicting the changes in hair count from baseline to 12 weeks in the 20 study participants P < 0.001, paired Student’s t-test

All patients successfully completed the study. Hair count significantly increased after 12 weeks of treatment with the lotion containing magnetized saline water; the mean hair count was 20.6 ± 9.8 at baseline and 32.5 ± 12.4 at 12 weeks (P < 0.001, paired Student’s *t*-test, Figure 1).

**Figure 2:**
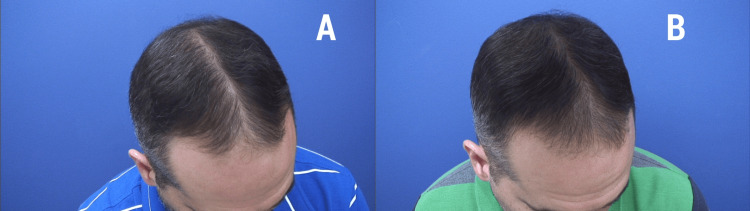
Illustrative images of the visible increase in hair growth observed from baseline (panel A) to 12 weeks (panel B) induced by a topical lotion containing magnetized saline water

Similarly, the mean HMI significantly increased from 37.8 ± 11.4 at baseline to 45.1 ± 13.6 at 12 weeks (P < 0.01, paired Student’s *t*-test). An illustrative example of the effect of the lotion is reported in Figure 2.

The treatment was well-tolerated, and none of the patients reported adverse local or systemic effects. Eight patients (40%) rated their overall satisfaction with the topical lotion as excellent, another eight patients (40%) rated it as good, two patients (10%) rated it as average, and two patients (10%) rated it as poor.

Molecular study

**Figure 3:**
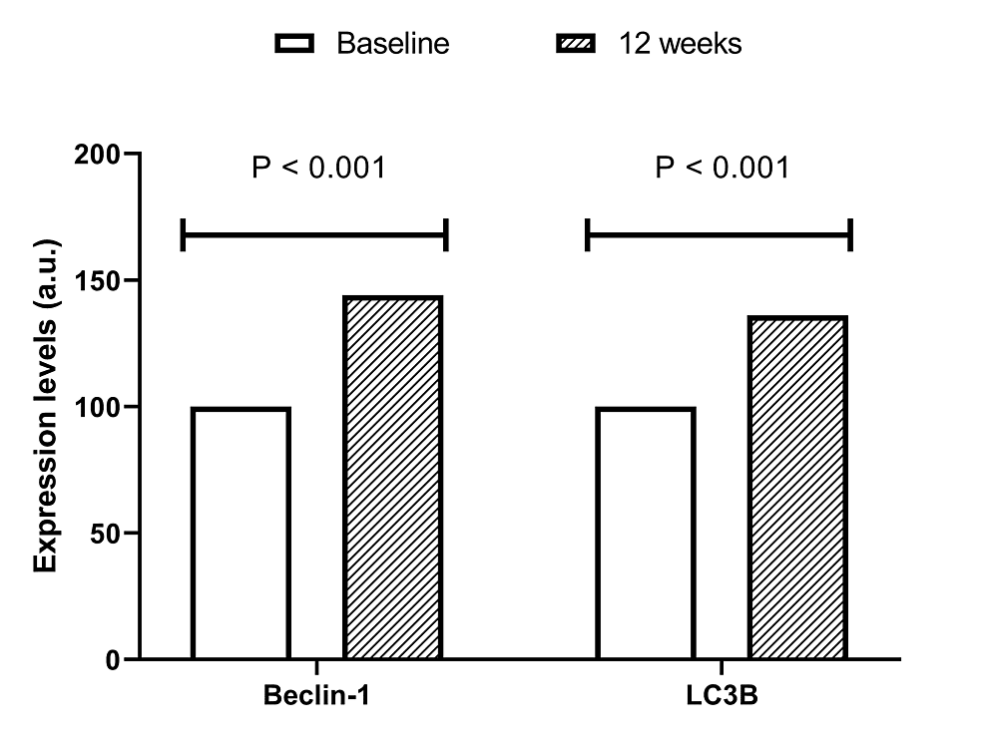
Clustered bar charts depicting the changes in scalp Beclin 1 and LC3B levels from baseline to 12 weeks in the 10 participants involved in the molecular study Both P < 0.001, paired Student’s *t*-test

The molecular study involving ten men revealed that the topical lotion effectively increased Beclin-1 levels in scalp biopsies by 44% at 12 weeks (144 a.u.; P < 0.001, paired Student’s t-test) as compared to baseline (arbitrarily set at 100 a.u.). In addition, LC3B concentrations increased by 36% from baseline (arbitrarily set at 100 a.u.) to 12 weeks (136 a.u., P < 0.001, paired Student’s t-test), consistently indicating that the lotion activated autophagy in the scalp (Figure 3).

Interestingly, a positive correlation was observed between the increase in both Beclin-1 and LC3B expression and the improvement in hair count and HMI (Spearman’s rho = 0.52, P < 0.05 for Beclin-1 and hair count; Spearman’s rho = 0.44, P < 0.05 for LC3B and hair count; Spearman’s rho = 0.46, P < 0.05 for Beclin-1 and HMI; Spearman’s rho = 0.40, P < 0.05 for LC3B and HMI).

## Discussion

This is, to our knowledge, the first clinical and molecular study to investigate the clinical potential of topical magnetized saline water in the management of mild-to-moderate androgenetic alopecia in men. From a clinical standpoint, we found that the application of the research product to the scalp resulted in a significant improvement in both hair count and HMI after 12 weeks of use. The lotion was well-tolerated, and users expressed a good overall satisfaction level. Mechanistically, the lotion effectively stimulated autophagy at the scalp level, as indicated by increased expression of Beclin-1 and LC3B at 12 weeks compared to baseline. Importantly, there were significant positive correlations between the increase in both Beclin-1 and LC3B expression in the scalp and the extent of improvement in hair density and HMI. These findings collectively suggest that the observed clinical effects of magnetized saline water on hair may be at least in part attributed to its ability to induce autophagy.

In a previous study by Parodi et al., it was observed that autophagy was actively occurring in hair matrix keratinocytes during the anagen phase of organ-cultured hair follicles [7]. Conversely, not only was autophagy found to be impaired during the catagen phase, but inhibiting autophagy actually promoted catagen development [7]. These findings suggest that one strategy for promoting hair growth in humans is to stimulate intrafollicular autophagy. We reasoned that topically applied magnetized saline water could act as a potential strategy to promote autophagy in the scalp because salinity-alkaline stress may exert autophagy-stimulating effects [15]. Another possibility is the local induction of hyperosmotic stress by magnetized saline water, which has been shown to upregulate aquaporin 3 (AQP3), a channel that primarily transports water, glycerol, and hydrogen peroxide [23,24]. AQP3 not only plays a crucial role in maintaining skin hydration, water retention, and barrier repair, but it also possesses the unique ability to directly bind to Beclin-1, thereby activating autophagy [25]. However, it remains to be answered whether the topical application of magnetized saline water enhances AQP3 expression. Despite the poorly understood mechanisms of autophagy activation by magnetized saline water, the significant positive correlations observed between the increase in Beclin-1 and LC3B expression in the scalp, along with the notable improvements in hair density and HMI, strongly indicate that the clinical effects induced by the lotion were primarily autophagy-dependent. However, we cannot exclude that magnetized saline water may also promote hair growth through autophagy-independent mechanisms. Accordingly, magnetic fields can modify conductivity, dielectric constant, pH, surface tension, and dissolved oxygen levels, leading to enhanced nutrient and gas supply to the scalp [11,18]. To fully harness the clinical benefits of magnetized saline water, further research is needed to clarify its mechanisms of action. When evaluating the benefits of an intervention for treating androgenetic alopecia, it is also important to consider the impact it has on patients. In our study, we found that 80% of participants rated the topical lotion as excellent or good, indicating a high level of satisfaction. However, it is crucial to conduct independent replication studies to validate and build upon these promising results.

Our findings should be interpreted within the context of several methodological limitations. Firstly, the clinical investigation was designed as an open-label study and did not include a placebo arm. This design may introduce bias as participants and researchers are aware of the treatment being given, which could potentially influence the results. Secondly, the study was limited to Caucasian men, and scalp biopsies were only performed on ten participants. This narrow demographic focus and limited biopsy sampling may limit the applicability of our findings to other ethnic groups and the broader population. Thirdly, our study had a small sample size (n = 20), which may further limit the generalizability of our findings. Fourthly, we did not include women, which prevents a sex-based analysis of the effects of topically applied magnetized saline water on hair growth. Given these limitations, it is important to note that our results should not be used as a basis for treatment recommendations. Patients with androgenetic alopecia should be treated on an individual basis, taking into account each patient's characteristics based on the results of well-designed clinical trials. To overcome these caveats, future research should consider a larger, more diverse sample size that includes both men and women of various ethnic backgrounds. A double-blind, placebo-controlled trial design could also help to reduce potential bias and increase the validity of the results. In addition, it would be beneficial to include a larger number of scalp biopsies to provide a more comprehensive understanding of the effects of the treatment on the autophagic flux. While autophagy is generally considered beneficial for hair growth, it is important to understand the optimal levels and control mechanisms to prevent any potential negative consequences.

## Conclusions

After 12 weeks of topical treatment, a lotion containing magnetized saline water activated scalp autophagy and significantly increased hair count and HMI in men with mild-to-moderate androgenetic alopecia. While the results of this study are promising, further research is needed to fully understand the mechanisms by which the lotion promotes hair growth and to confirm its long-term safety and efficacy.
